# Husbands involvement in birth preparedness and complication readiness in Axum town, Tigray region, Ethiopia, 2017

**DOI:** 10.1186/s12884-019-2338-z

**Published:** 2019-05-22

**Authors:** Zeray Baraki, Fthi Wendem, Hadgu Gerensea, Hafte Teklay

**Affiliations:** 1 Department Nursing, Aksum University Health Science College, Axum, Ethiopia; 2 Department Biomedical, Aksum University Health Science College, Axum, Ethiopia

**Keywords:** Husband involvement, Birth preparedness, Complication readiness

## Abstract

**Background:**

Worldwide, around 289,000 maternal mortalities occur each year from complications related to pregnancy and childbirth. In Ethiopia, the maternal mortality rate is among the highest in the world. This is mainly contributed by the three delays. Husbands’ involvement in birth preparedness and complication readiness can significantly reduce these delays. Therefore, it is important to know what, currently, is the husbands view regarding knowledge of key danger sign and involvement about birth preparedness and complication readiness. The investigation of husband’s involvement in birth preparedness and complication readiness was sounded throughout much of the developed world. However, despite the putative assumption that the problem exists in Ethiopia at large and Axum in particular, there is no article related to the problem as yet. For this reason, the study intended to assess husband’s involvement in birth preparedness and complication readiness.

**Method:**

A community based cross-sectional study was conducted in Axum Town from September–June 2017. Data were collected from a randomly selected sample of 406 husbands using the lottery method and interviewer administered structured questionnaires. Data were entered into Epi info version 7 and analyzed using SPSS version 20. The statistical analysis was made at the 95% confidence level. The data were summarized and described using descriptive statistics.

**Result:**

Four hundred six husbands were participating in the study with a response rate of 98%. Out of total respondents 258(64.7%) were in the age range of ‘18 - 39’ years and the mean age was 36.55 years. Three hundred forty-three (86%) of the respondents has attended primary education or above and 187(46.9%) fulfilled five or more variables of parameter for husband’s involvement in birth preparedness and complication readiness and leveled as having good involvement. One hundred sixteen (29.1%) respondents had gotten health service problems that prohibited from going to the health facility.

**Conclusion:**

Based on the findings, the overall involvements by husbands in birth preparedness and complication readiness were low. Most of the respondents have low knowledge. Knowledge of husbands, health service issues, facility and quality of care are reported factors that affect husband’s involvement in birth preparedness and complication readiness.

**Electronic supplementary material:**

The online version of this article (10.1186/s12884-019-2338-z) contains supplementary material, which is available to authorized users.

## Background

Worldwide, around two hundred eighty-nine thousand maternal mortality occurs each year from complications related to pregnancy and childbirth of which 99% has occurred in resource limited countries. Sub-Saharan Africa had taken the highest maternal mortality rate (MMR) [1]. Even though, a number of countries have established several ways of reducing child mortality rate, maternal and neonatal mortality still remains a huge public health problem [2, 3].

Based on World Health Organization (WHO) inference global maternal mortality decreased from 523,000 to 289,000 within a time interval of 1999–2013, which accounts for 45% reduction. In Sub-Saharan countries, in the same time period, the maternal mortality was reduced from 990,000 to 510,000 with a 49% reduction. Similarly, in Ethiopia the maternal mortality was 29,000 in 1990, and this was reduced by 37.9% to 11,000 by the year 2015 [4, 5].

The most common cause of maternal mortality in Ethiopia was a maternal problem, notably, obstructed labor and postpartum bleeding. But equal consequences for the survival of women during pregnancy, delivery and post-natal period by preventing maternal related morbidity is access to skilled birth attendant and emergency obstetric care [6].

In 2014 WHO reported that the MMR in Ethiopia was 420 per 100,000 live births which was much higher than the universal maternal mortality [5]. The major direct causes of maternal mortality in Ethiopia are ruptured uterus (12%), preeclampsia/eclampsia (11%), obstructed/prolonged labor (13%), malaria (9%) and abortion (6%) [7]. These maternal health problems have been prevented success through expanding quality maternal health services within the community and avoiding the three delays during pregnancy, which was delayed in seeking skilled emergency obstetric care, delay in reaching the health facility and delay in receiving timely and effective intervention after reaching the facility [8, 9].

Despite all the progress over the year, in most developing countries, including Ethiopia, maternal mortality still continues to be an enormous challenge. The most contributing factor to this problem is families do not take timely action; the family tries to take steps only when labor begins. When complications occur, the unprepared family invests a great deal time in handling the problem, searching the source of money, finding transport and reaching the right referral facility. Over all these delays may increase the occurrence of maternal mortality [10].

The decline in maternal death in Ethiopia is below the average compared to both Sub- Saharan countries and the worldwide decline. In addition, while several countries have met the millennium development goal (MDG), Ethiopia has not achieved the set MDG target for MMR. The development goal and target were set with the purpose that it would press countries put more efforts, identifying the barriers to qualified maternal health services as well as addressing at all levels of the health system to contribute for the achievement of sustainable development goal (SDG). In order to meet the SDG target, Ethiopia has developed a five-year plan from the 2016/17 with the target of reducing maternal mortality rate from 420 to 199 per one hundred thousand live births. Moreover, in the same time period, there is also a set target of making 90% husband to be involved in birth preparedness and complication readiness (BPCR) [11]. There are different factors which affect the husband’s involvement in birth preparedness and complication readiness, of which knowledge, health and facility service factor, communication issue and cultural factor are the most common factors [12–14].

Birth preparedness and complication readiness have been considered as a comprehensive strategy aimed at promoting a safe motherhood. Husband involvement in reproductive health has also been promoted as a promising new strategy for improving maternal and child health through addressing the three delays during pregnancy. Considering the potential positive impact of the involvement, it would be of paramount importance to evaluate the current husband trend regarding knowledge on key danger sign and involvement about birth preparedness and complication readiness. The investigation husband’s involvement in BPCR was assessed in much part of the world, despite the putative impact of husband’s involvement, there is, to our best knowledge, no research outcome regarding the problem in the Axum town [15–18]. For this reason, this study was intended to assess husband’s involvement in birth preparedness and complication readiness in the Axum town Tigray region, North Ethiopia, 2017.

## Methods

### Study area and period

The study was conducted from September 2016 to June 2017 in Axum town, Tigray regional, state North Ethiopia, located about 1041 km North of Addis Ababa.

### Study design

A community based cross-sectional quantitative study design was conducted among husbands whose wives had an infant of less than 12 months old in Axum town.

### Source population

Husbands whose wives’ had an infant of less than 12 months old in a community household.

### Study population

Sampled husbands whose wives had an infant of less than 12 months old in a community household.

### Inclusion and exclusion criteria

#### Inclusion criteria

Husbands whose wives had a child less than 12 months old and permanent residents (lived for at least 6 months in Axum town),

#### Exclusion criteria

Husbands who were not staying together with their wives during pregnancy and birth of the child and those who were critically ill.

### Sample size determination

#### Sample size

The minimum required sample size was calculated by considering the single population proportion based on the following assumptions. A study conducted in Mekelle town in 2014 showed that about 60% of the husbands had participated in birth preparedness and complication readiness [12]. A level of confidence of 95%, margin of error of 5% and the non-response rate of 10% were also considered. With this the final sample size became 406 husbands.

Sample size:- .$$ n=\frac{{\left(z\frac{a}{2}\right)}^2p\left(1-p\right)}{d^2} $$

Where *n* = estimated sample size.

*P* = prevalence.

D = marginal error

$$ n=\frac{\kern0.2em {(1.96)}^2\times 0.60\kern0.2em \left(1-0.60\right)\kern0.1em }{(0.05)^2}=368.8 $$

Non response rate 10%.

Then the total sample size is **406.**

#### Sampling methods and procedures

**Fig. 1:**
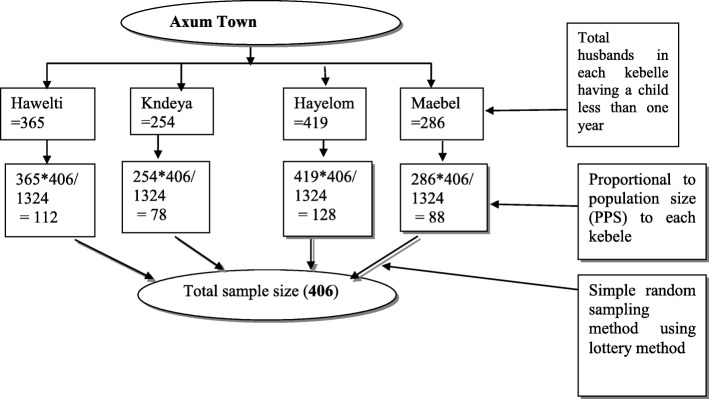
Schematic representation of sampling procedure

Axum town has 4 kebeles and all of them were included in the study. Initially, a census was conducted to register all households having under-1 year child. Accordingly, the number of husbands whose wives had less than 12 month’s old infant in each kebele was: 365, 254, 419 and 286 for Hawelti, Kndeya, Hayelom and Maebel kebeles respectively. After proportion allocation of the sample among the kebeles was employed, the sampling frame was prepared. From the sampling frame, 406 participants were selected by simple random sampling proportionally for each kebele. Since participants have the right to decline and be considered as non-respondents, those participants who declared their wish not to participate were treated as neutral. (Fig. 1).

### Variables of the study

#### Dependent variable

- **Husband involvement in BPCR**

#### Independent variables

##### Demographic and socio-economic characteristics

**Table:** 

Age,	Income
Religion,	Occupation
Level of education	Income earner

##### Knowledge of husbands on key danger sign and Health services factor

**Table:** 

Key danger signs during pregnancy	Health service problems
Key danger signs during labor	Quality care
Key danger signs during postpartum	Means of transportation

### Operational definitions

**Birth preparedness and complication readiness practice among husbands**:**-** is a strategy to encourage husbands to be informed of the danger signs of obstetric complications and emergencies, choose a preferred birth place and attendant at birth, arrange for transport to the skilled care site in case of an emergence, saving or arranging alternative funds for costs of emergency care, and accompany her to emergency care. Identifying a blood donor, preparing clean clothes for the mother /mother’s baby and arrange a source of household support to provide temporary family care during her absence [10].

**Good involvement in BPCR**:- are those who practiced at least five components from eight parameters of BPCR [18].

**Poor involvement in BPCR**:- are those who practiced less than five components from eight parameters of BPCR [18].

**Knowledge of key danger signs of pregnancy:-** A husband is considered knowledgeable if he spontaneously mentioned all the three key danger sign of pregnancy, such as vaginal bleeding, blurred visions and swollen hands/face otherwise not knowledgeable [10].

**Knowledge of key danger sign of labor:-** A husband is considered knowledgeable if he spontaneously mentioned all the four key danger sign of labor such as severe vaginal bleeding, convulsions, prolonged labor and retained placenta otherwise not knowledgeable [10].

**Knowledge of key danger sign of postpartum:-** A husband is considered knowledgeable if he spontaneously mentioned all the three key danger sign of postpartum such, as severe vaginal bleeding, foul smelling and high fever otherwise not knowledgeable [10].

### Data collection procedure

Data were collected through a structured self-administered questionnaire and interview. The data collection was collected by employing trained nurses under supervision of the principal investigator and trained supervisor from April 1 to May 2, 2017.

### Data collection tools

Interview-administered questionnaire was adapted from the safe motherhood questionnaire developed by maternal and neonatal health programs of JHPIEGO which contains five main parts. Part I about Basic demographic information, part II about Knowledge on key danger sign among husbands, part III about Husbands involvement/practice in BPCR, part V about Health service factor and part VI about communication issue [16]. An additional data file shows this in more detail. **(see** Additional file 1**).**

### Data quality assurance

Data quality was assured through pretest of the questionnaire and necessary corrections were made. The pretest was done among 20 husbands in Adewa town. The English questionnaire was translated into local language Tigrigna and retranslated back into English by experts. Filled out questionnaires was checked for completeness by supervisor cleaning, coding and entering of the data were carried out carefully. The study was considering the validity of data with a limitation of recall bias that the husbands had remembered with a maximum of 12 months back.

### Data processing and analysis

After checking the data for its completeness, missing values and coding of questionnaires, data were entered into Epi info version 7 and analyzed using SPSS version 20. The statistical analysis was made at the 95% confidence level and with a 5% margin of error. The data were summarized and described using descriptive statistics. Descriptive statistics such as proportion, Percentage, ratios, frequency distribution, mean, and standard deviation was used to describe the data and to explain the findings.

### Ethical considerations

Ethical clearance was obtained from the Institutional Review Board committee of the Aksum University College of Health Sciences. The Institutional Review Board committee concluded that the community educational characteristics data, which was taken from city administration before data collection, indicate that part of the community did not attend formal education and could not read. Data collectors read the informed consent and explained the purpose of the study, how participants were selected, and the risk and benefit to help women decide on participation. Each study participant was also informed about confidentiality, privacy, and the right to withdraw or stop the interview throughout the whole interview process.

### Dissemination of results

The final report was presented to Aksum University Health Science College, and a copy of it was offered to the Axum town health bureau and regional health office. Efforts were made to publish the findings in local or international journals. Attempts were made to present the findings of this study in national and international scientific conferences.

## Results

### Demographic and socio-economic characteristics

Out of the 406 sampled husbands, 399 responded to the interview with a response rate of 98%. Out of total respondents 258(64.7%) were in the age range of ‘18 - 39’ years and the mean age was 36.55 years. Three hundred forty-three (86%) of the respondent has attended primary education or above. Two hundred forty-six (61.7%) were Orthodox in religion and 323(81%) were employed in the government, non-government and Private organization. **(**Table 1**).**

**Table 1:** Demographic and socio economic characteristics of husbands in Axum Town, Tigray Region, Ethiopia, 2017, (*N* = 399)

Variables	Category	Frequency	%
Age in year	18–29	82	44.1
	30–39	176	24.8
	40–49	99	10.5
	> = 50	42	20.6
Religion	Orthodox	246	61.7
	Muslim	145	36.3
	Others^a^	8	2.0
Education status	No formal education	56	14
	Primary [1–8]	90	22.6
	Secondary [9–12]	107	26.8
	Higher(12+)	146	36.6
Occupation	Merchant	40	10.3
	Employed (GO and NGO)	159	39.8
	Private employed	157	39.3
	Others^b^	36	9.0
Monthly income	< 500	27	6.8
	500–1000	103	25.8
	> 1000	269	67.4
Income earner	Husband only	223	55.9
	Wife’s only	13	3.3
	Both husband and wife	163	40.9

Others^a^:- Catholic & Protestant. Others^b^; Daily labor, Farmer & Students. GO; Governmental. NGO; Non Governmental

### Husbands knowledge on key danger sign during pregnancy, labor and postpartum

#### Knowledge on key danger sign during pregnancy

Among the study subjects 213(53.4%), 117(29.3%) and 165(41.4%) respondents spontaneously mentioned vaginal bleeding, blurred visions and swollen hands/face, respectively, as a key danger sign during pregnancy. While 293 (73.4%) of the respondents spontaneously mentioned at least one and above key danger sign, 152(38.1%) respondents mentioned at least two and above key danger sign and 50(12.5%) respondents mentioned all the three key danger signs.

#### Knowledge on key danger sign during labor/delivery

Among the study subjects 264(66.2%), 163(40.9%), 169(42.4%) and 120(30.1%) respondents spontaneously mentioned severe vaginal bleeding, convulsions, prolonged labor and retained placenta, respectively, as a key danger sign during labor/delivery. While 332(83.2%) of the respondents spontaneously mentioned at least one and above key danger sign, 227(56.9%) and 100(25.1%) respondents respectively mentioned at least above two and three key danger sign. Lastly all the four key danger sign was mentioned by 57 (14.3%) respondents.

#### Knowledge on key danger sign during postpartum

From all respondents, about, 137(34.3%), 64(16%) and 84(21.1%) respondents spontaneously mentioned severe vaginal bleeding, foul smelling and high fever respectively, as a key danger sign during postpartum. One hundred eighty-four (46.1%) of the respondents spontaneously mentioned at least one and above key danger sign, 71(17.8%) respondents mentioned at least two and above key danger sign and 30(7.5%) respondents mentioned all the three key danger signs. **(**Tables 2 and 3**).**

**Table 2:** Knowledge of husbands on key danger sign during pregnancy, labour, post partum and in Axum Town, Tigray Region, Ethiopia, 2017 (*N* = 399)

Knowledge of key danger sign	Frequency	%
The three key danger sign during pregnancy		
vaginal bleeding	213	53.4
blurred visions	117	29.3
swollen hands/face	165	41.4
The four key danger sign during labor/delivery		
severe vaginal bleeding	264	66.2
Convulsions	163	40.9
prolonged labor	169	42.4
retained placenta	120	30.1
The three key danger sign during postpartum		
severe vaginal bleeding	137	34.3
foul smelling	64	16
high fever	84	21.1

**Table 3:** Summery of knowledge of husbands on key danger sign during pregnancy, labour, post partum in Axum Town, Tigray Region, Ethiopia, 2017 (*N* = 399) . Continued

Knowledge of key danger sign	Frequency	%
Husbands knew at least one and above key danger sign during pregnancy	293	73.4
Husbands knew at least two and above key danger sign during pregnancy	152	38.1
Husbands knew all three key danger sign during pregnancy	50	12.5
Husbands knew at least one and above key danger sign during labor/delivery	332	83.2
Husbands knew at least two and above key danger sign during labor/delivery	227	56.9
Husbands knew at least three and above key danger sign during labor/delivery	100	25.1
Husbands knew all the four key danger sign during labor/delivery	57	14.3
Husbands knew at least one and above key danger sign during postpartum	184	46.1
Husbands knew at least two and above key danger sign during postpartum	71	17.8
Husbands knew the three key danger sign during postpartum	30	7.5

### Level of husbands involvement in birth preparedness and complication readiness

Eight variables were used to measure the status of husbands’ involvement in BPCR. Of all respondents, 29.3% had identified the skilled birth attendant, and 54.6% identified a preferred birth place and attend at birth, 59.4% arranged household support to provide temporary family care during her absence, 44.6% arranged for transport to the skilled care site in case of emergence and 39.8% had accompanied her to emergency care. Similarly, 47.6% arranged blood donor, 39.6% arranged alternative funds for costs of emergency care and 86.5% prepared clean clothes & other material for the mother and newborn baby. From these eight parameters, husbands fulfilling five or more were labeled as having **“Good involvement”** and otherwise **“Poor involvement**”. Accordingly, 187(46.9%) fulfilled five or more variables and leveled as having good involvement. **(**Table 4**).**

**Table 4:** Birth preparedness and complication readiness practice for husbands in Axum Town, Tigray region, Ethiopia, 2017

Variables	Yes	%
Identified skilled birth attendance	117	29.3
Identified a preferable birth place	218	54.6
Arrange household support	237	59.4
Identified transportation	178	44.6
Personally accompanied	159	39.8
Blood made ready/donated	190	47.6
Saved money for costs of skilled and emergency care	158	39.6
Prepared clean clothes & other material	345	86.5
Over all husbands involvement in BPCR (fulfilled > = 5)	187	46.9

#### Health service issues

**Fig. 2:**
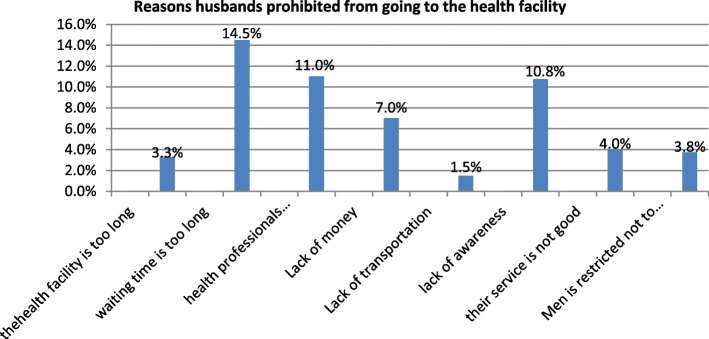
Reasons of the respondents prohibited from going to the health facility in Axum Town, Tigry Region, Ethiopia 2017(*N* = 399)

Of all respondents, 283 (70.9%) husbands had not gotten any health service problems that prevented from going to the health facility, the rest 116 (29.1%) had gotten health service problems that prohibited from going to the health facility. The reason that discouraged respondents from going to a health facility has reported accordingly. Thirteen (3.3%) of respondents reported that the health facility is too long, and 58 (14.5%) of respondents reported that waiting time to get service is too long, 44(11%) reported that the health professional approach is not good, 28(7%) reported that lack of money, 6 (1.5%) reported that lack of transportation, 43(10.8%) reported that lack of awareness, 16(4%) services is not good and 15(3.8%) men is restricted not to enter to labor room. **(**Fig. 2**).**

#### Quality care

Three hundred forty-four (86.2%) of the respondents reported that the service gave in the health facility was good. But the rest 55 (13.8%) of the respondents reported that the service given in the health facility was bad. The respondents mention some of the reasons for bad services in the health facility as follows; 37 (9.3%) reported that the health care providers didn’t respect me, 35 (8.8%) reported that the waiting room is not good, 34 (8.5%) reported that the waiting time is too long and 4(1.0%) reported that there is no private room for examinations.

## Discussion

This study identified different elements in the husband’s involvement in birth preparedness and complication readiness. Within this context, identification of skilled birth attendance, Identified a preferable birth place, Arrange household support, transportations, personally accompanied their wives, blood made ready/donated when complications happened, Saved money for costs of emergency care and prepared clean clothes and other materials for the mother/mother’s baby are very important for the involvement of husbands in birth preparedness and complication readiness.

This study showed that nearly half, 46.9% of husbands were involved in birth preparedness and complication readiness, for at least five of the eight components. Compare to a similar study done in Mekelle, 2014 which had a 60.4% involvement, the result found here was significantly lower. This higher difference may have stemmed from the difference in performance for similar interventions in the same region. But, the result of this study was congruent with the result of similar study done in Tulsipur, Nepal in 2011 which revealed that 44.37% of husbands were involved in birth preparedness the similarity might be similar strategy on safe motherhood may be occurred in the two countries [18, 19].

In this study, about 23.9% of husbands identified skilled birth attendance. This low level also was observed in a study done in Enderta woreda in 2012 year, which revealed that 32.70% husbands identified skilled birth attendance [3]. This similarity could be from similar intervention was made in the same geographical area. But it is lower than study in Kathmandu 47.9%, in Tulsipur, Dang District Nepal 51.8% and South-west Uganda 91.1% respectively of men arranged skill birth attendant. This discrepancy might be from the difference in accessibility of a skill birth attendant [2, 15, 19].

This study also identified that, 39.6% respondents saved money for costs of emergency care; similar result was found in a study done in rural Uganda in 2010, in which 44.3% respondents saved money for emergency complications. In another similar study, the financial saved was lower than a study conducted in Yangon, Myanma in 2017, which showed that 81.7% respondents saved money for complications. This might be through the difference in socio, demographic factor [2, 14]. In this study the numbers of raised factors are limited to fit the assumption of logistic regression to do the association between dependent and independent variable.

In this study, nearly half of the respondents were involved in accompanying their wives, identifying transportation and readying blood ahead of childbirth, which accounts 39.8, 44.6 and 47.6%, respectively. Similar findings were obtained in a study conducted in Nepal Kathmandu in 2010, in which 39.3% respondents accompanying their wives for ANC, 30.2% arranged transportations [15]. But relatively higher result was seen in Northern Nigeria 2010, in which 18.7% respondents accompanying their wives to the hospital, 3.7% prepared blood in case of complications and 24.2% identified for transportation. These variations could be by difference in cultural practices, poverty and in effective implementations of safe motherhood in different countries of health system [13].

## Conclusion

Out of the 406 study subjects, 399 responded to the interview making a response rate of 98%. Based on the findings, the overall involvements by husbands in birth preparedness and complication readiness were low. Most of the respondents have low knowledge on identifying all key danger sign during pregnancy, delivery/labor and postpartum. Knowledge of husbands, health service issues, facility and quality of care are reported factors that affect husband’s involvement in birth preparedness and complication readiness.

## Additional files

Additional file 1: Information sheet and questionnaire of the study. It is a data contained the information sheet for informed consent and a questionnaire of the study. (DOCX 24 kb)

Additional file 2: It is a dataset of the study in excel format. It is a data contained the detail dataset on excel format used for analysis and interpretation of the study. (XLSX 77 kb)

## Abbreviations

BPCR: Birth preparedness and complication readiness
EDHS: Ethiopia demographic and health Survey
FMOH: Federal ministry of health
JHPIEGO: Johns hopkins program for international education in gynecology and obstetrics
MDGs: Millennium development goals
MMR: Maternal mortality ratio
SDG: Sustainable development goal
