# Supplementary material for: Husbands involvement in birth preparedness and complication readiness in Axum town, Tigray region, Ethiopia, 2017
Source: BMC Pregnancy Childbirth. 2019 May 22;19:180. doi: 10.1186/s12884-019-2338-z (PMC6530057; doi:10.1186/s12884-019-2338-z)
Supplement: Supplementary file 1 — Information sheet and questionnaire of the study. It is a data contained the information sheet for informed consent and a questionnaire of the study. (DOCX 24 kb) [file 12884_2019_2338_MOESM1_ESM.docx]

**Information sheet**

I am coming from Aksum University. We are carrying out a study on husband’s involvement in birth preparedness and complication readiness (BPCR) in Aksum town.

**Purpose of the study**; we would like to find out the reasons why most husbands in the town are not actively involved in BPCR especially regarding accompanying their partners to the health unit to seek antenatal care, delivery and postnatal care services. The study will provide an opportunity to obtain a comprehensive picture on husband’s involvement in BPCR.

**Benefits of the study: -** The Findings of the study will provide information on how best to involve men in BPCR and to improve the health of both men and women as a whole. Study participants who may have any medical illness will receive free consultation services from the research team and where it requires referral they will be referred to the health facilities for the management of their conditions

**Risks of the study: -**No invasive procedures during the study. However some of the questions I will ask sensitive and a private nature, and may make you feel uncomfortable. Please be assured that this discussion is strictly confidential, and names will not be recorded. The findings of the study will be generalized and not linked to the individual.

**Participation in the study: -** Participation in the study is voluntary, if you do not want to answer particular questions that are okay. There are no rights or wrong answers to the questions that we will be asking you. Please feel free to answer exactly as you feel. You are free to withdraw from the interview at any time without the need to justify your decision.

**Consent Form**

I would like to seek your consent before I proceed. Based on the above information are you willing to participate in this study?

1. Yes 2. No

If the Respondent agrees to continue, ask if he has any questions. Respond to the questions as appropriate, and then start discussion.

If the Respondent does not agree to continue, thank him/her and go to the next interview.

Data collector: Name Signature _______ Date ___________

Interviewed signature Date

Supervisor: Name Signature _________ Date ___________

##

##

**Questionnaire**

English Version Questionnaire

Results of interview questionnaire

1. Completed 2. Refused 3. Partially completed

| Respondents identification  001. Questionnaire Code______ 002. Respondent’s Kebelle ____________________  003. House No ____________  **Instruction: Circle the appropriate answer** provided and where applicable writes the required responses in the spaces provided.  **Section 1: Basic demographic information** | | |
| --- | --- | --- |
| **S.N** | **Questions** | **Answers** |
| 101 | How old are you? | Age in years:_____________ |
| 102 | What is your religion? | 1. Orthodox 2, Muslim 3, Catholic 4, Protestant  5. Other (specify)………………… |
| 103 | Educational level | 1, No formal education 2, Primary (1-8) 3, Secondary (9-12) 4, Higher (12+) |
| 104 | What is your present occupation? | 1. Government employed 2. Private/own employed  3. NGO employed 4. Merchant 5.Student 6. Farmer  7. Daily laborer 8. Other (specify) _______ |
| 105 | What is your average monthly income in birr? | 1, <500 2, 500 – 1000 3, >1000 |
| 106 | Who is the income earner in your family? | 1, Only husband 2, Only wife 3, Both husband and wife 4, Neither husband nor wife |
| **Section 2: Knowledge on key danger sign among husbands** | | |
| 201 | In your opinion, can unforeseen problems related to pregnancy or child birth occur that could endanger the life of a woman? | 1. Yes 2. No 3, I don’t know |
| 202 | From where did you hear the information about some serious health problems that can occur before, during and after delivery? More than one answer is possible | 1. Hospital 2. Health center 3. Relatives 4. Health extension workers  5. Radio, TV  6. Other______________ |
| 203 | In your opinion, what are some serious health problems that can occur **during pregnancy** that could endanger the life of a pregnant woman?  PROBE: Any others? And encircle all that the husband response (multiple answer is possible) | 1. Bleeding  2. Severe headache  3. Blurred vision  4. Convulsions  5. Swollen hands/face  6. High fever  7. Loss of consciousness  8. Difficulty breathing  9. Sever weakness  10. Severe abdominal pain  11. Accelerated/reduced fetal movement  12. Don’t know any  13. Other Specify __________ |
| 204 | In your opinion, what are some serious health problems that can occur **during labour and child birth** that could endanger the life of the woman?  PROBE: Any others? | 1. Severe Bleeding  2. Severe headache  3. Blurred vision  4. Convulsions  5. Labour lasting >12 hours  6. High fever  7. Loss of consciousness  8. Retained placenta  9. **Don’t know any** |
| 205 | In your opinion, what are some serious health problems that can occur **during the first 2 days after birth** that could endanger the life of the woman?  PROBE: Any others? | 1. Severe Bleeding  2. Severe headache  3. Blurred vision  4. Convulsions  5. Swollen hands/face  6. High fever  7. Loss of consciousness  8. Difficulty breathing  9. Sever weakness  10. Malodorous vaginal discharge  11. Other Specify __________  12. **Don’t know any** |
| 206 | In your opinion, what are some serious health problems that can occur **during the first 7 days after birth** that could endanger the life of a newborn baby?  PROBE: Any others? | 1. Difficult or fast breathing  2. Yellow skin color (Jaundice)  3. Poor sucking/feeding  4. Pus, bleeding or discharge around umbilical cord  5. Baby very small  6. skin lesion or blisters  7. Convulsions/spasms/rigidity  8. Lethargy/unconscious  9. Red or swollen eyes with pus  10.Other Specify __________  11. **Don’t know any** |

**Section 3: Husbands involvement/practice in BPCR**

| 301 | Had you identified a skilled birth attendance for your wife’? | 1. Yes 2. No |
| --- | --- | --- |
| 302 | Had you identified a preferable birth place and attendant at birth? | 1. Yes 2. No |
| 303 | Did you arrange a source of household support to provide temporary family care during her absence? | 1. Yes 2. No |
| 304 | Had you identified means of transportation for your wife’s? | 1. Yes 2. No |
| 305 | Had you saved or arranged alternative funds for costs of skilled and emergency care? | 1. Yes 2. No |
| 306 | Had you accompanied your wife to go to health facility? | 1. Yes 2. No |
| 307 | Had you planned to donate blood when complications happened? | 1. Yes 2. No |
| 308 | Had you prepared clean clothes & other materials for baby/mother’s? | 1. Yes 2.No |
| **Section 4: Health service factor** | | |
| 401 | How much time do you spent to go to the health facility from your home? | ________________ Minutes |
| 402 | What means of transport do you use to get to the health facility? | 1. By foot 2. Public transport (Bus, tax and motorcycle) 3. Ambulance 4. Private vehicle  6. Others (specify)…………………… |
| 403 | Are there any health service problems that you are facing that prevented you from going health facility? | 1. Yes 2. No |
| 404 | What problems that may prevent or discourages you from going health facility. | 1. Distance to the health facility is too long  2. I heard the waiting time to get service is too long  3. I heard the health professionals approach is not good  4. Lack of money 5. Lack of transportation  6. lack of awareness  7. I heard their service is not good  8. Men is restricted not to enter into labour room  9. Others specify ________________________ |
| 405 | How would you describe the service of the health facility during your wife delivered and afterwards? | 1. Very good 2. Good  3. Moderate 4. Bad 5. Very bad |
| 406 | What factor may make the health service bad/very bad? | 1.The waiting time is too long  2.There is no private room for examination  3. The waiting room is not good  4. The health care providers do not respect me  5. Other specify ______________________ |
| 407 | Would you recommend or encourage other husbands to go with their wives to this health facility? | 1. Yes 2. No |
| 408 | Do you have any comments to improve the quality of services of the Health facility? | 1. Yes  2. No |
| 409 | If your answer for question 408 is yes, What is your comment? | Specify ______________________________________________________________________________ |
| 410 | Who makes the final decision of where your wife is to deliver? | 1. Husband only 2.Wife’s only 3. Both husband and wife 4. Other specify ________________ |
| 411 | Are you as a man supposed to be involved in BPCR? | 1. Yes 2. No |
| 412 | If yes in question 411 above, in what way are you supposed to be involved? | Specify _______________________________________________________________________________ |

**Section 5; Communication issues**

| 501 | When you have problems who do you share it with first? | 1. My wife 2. My family (Brothers, Sisters or parents) /friend 3. Others (specify) ________________________ |
| --- | --- | --- |
| 502 | Have you ever received an invitation letter from a health worker inviting you to discuss pregnancy issues of your wife? | 1. Yes 2. No |
| 503 | Have you go to health facility to discuss with health professionals? | 1. Yes 2. No |

**Thank you very much for letting me talks with you today!!!**

**Thank you for participation!!!**
